# Cases of Tetanus

**Published:** 1809-09

**Authors:** J. Howship

**Affiliations:** Surgeon


Cases of Tetanus; by Mr. J. Howship, Surgeon.

*A Case of Tetanus, arising from a Wound, in which the Exhibition of Mercury was unsuccessful.*

——— Landers, private in the 13th Regiment of Foot, aged 23, received a slight wound in the sole of the foot, by treading upon a rusty nail. The accident happened on February 21, 1805, during the time of the regiment being upon garrison duty at Gibraltar.

For the first three days he suffered no pain from the wound; it was near the base of the great toe, and by his own account, it appeared to be very doubtful whether the point of the nail had penetrated beyond the common integuments; however, as he had reported himself, he was ordered to lay up his leg, and keep himself quiet, the only application to the wound being a mild dressing.

Upon the 4th day, the puncture became sore, tender, and inflamed; warm and emollient applications therefore were directed to be kept constantly applied to the foot; fomentations and poultices were alternately laid over the whole of the foot during the 5th, 6th, and 7th days.

On the morning of the 8th day, at the time of the poultice being removed, he felt and complained of a pain darting across his breast and about his jaws. These darts of acute pains came suddenly, as a shock of electricity, and were away again instantly; yet he described a constant but dull sort of pain, which he felt about the sides of the neck, and the angles of the jaws.

He made no complaint of severe pains in the back part of his neck; the tendency to spasm at this period seeming to prevail more in the lateral than the posterior muscles cles of the neck. From this time forward, his situation became more distressing, every hour adding to the severity of his sufferings.

On the first mention of pains about the neck and jaws, there being reason to suspect that the fascia of the foot had been wounded, the integuments and the tendinous expansion beneath were at once freely divided by the scalpel, cutting upon a director; by this means the wound was increased to nearly an inch in length. A fermenting poultice was laid upon the parts, and was renewed every twelve hours.

During the course of the disease, in this instance, the skin was ever free and perspirable, the excessive discharge by sweating only came on after the dreadful pains of the spasms had "run through his body." These pains always commenced from below near the umbilicus, and about the region of the false ribs, passing upwards upon the chest, and reaching as far as the insertion of the muscles of the neck into the mastoid process of the temporal bone.

The patient was himself of opinion, that the laying open the wound had produced some change in the situation and severity of the pains; he neither felt so much, nor so intense a degree of pain about his throat, as he had experienced prior to the division of the fascia. The pains had become more universal, shooting about the shoulders and neck more than formerly, although attended with a less severe impression upon the chest.

He frequently said he felt that his heart was at times ready to break with the distress of its pain. During intervals, however, he was nearly, or altogether free from pain, and expressed himself as being quite comfortable; at such times he would drop asleep for a quarter of an hour, till from some unfortunate attempt at motion, he was suddenly roused from his slumber, to endure the most excruciating torment.

When attacked in this way, his impatient desire was always to be laid as quickly as possible upon his back; he found this posture agree with his complaint better than any other, probably, because it in some measure operated as a check to the excessive contraction of the numerous and powerful assemblage of muscles situated posterior to the spine.

He found that the pains most particularly relieved by resting on his back, were those that passed across the breast, and towards the shoulder blades. In the muscular parts of the neck he almost constantly felt more or less of a dull a dull irksome pain, as well as at the angles of the jaw, where the permanent stiffness, rather than the degree of pain, distressed him.

Upon the 11th day, ʒij. of mercurial ointment were rubbed in upon the back and loins. The bowels were occasionally regulated by a laxative enema. The regular and natural state of the pulse was not at any time altered, even during the most severe paroxysms of pain. He received sufficient support in soups and sago with wine.

On the 12th day, a second consultation was held, the result of which was a second and more extensive division of the wounded parts.

The scalpel was again passed freely through the wounded integuments, and the incision was carried considerably deeper than the fascia, the perfect division of which was the object held principally in view.

The wound did not bleed three drops, and the man, though he felt intense pain at the time of the operation being performed, soon became easier; but he still suffered from occasional returns of spasm, which always commenced down at the groin, flying upwards to the breast and neck. ʒij. of mercurial ointment were rubbed in during the fore part of this day also.

Three hours after the enlargement of the wound a bleeding came on; the hæmorrhage was both sudden and considerable, he lost about ℥viij. of blood.

It is very worthy of observation, that the moment the bleeding commenced "he found his jaws become much looser than before," and they remained in this state of partial release till the time of his decease.

Upon the 13th day, this poor man was greatly altered, and much reduced in strength. He said in the forenoon that he was better, but that he was very weak; his pulse was remarkably lower than on the preceding day; the alteration was so great, as hardly to be explained by the quantity of blood lost by hæmorrhage the day before. Indeed the pulse was so much sunk as to raise doubts as to the possibility of his recovery.

He still, however, took wine and other nutriment in the most convenient forms, and opened his mouth sufficiently well to admit the passing a table-spoon. He said the cramps were certainly less severe than they had been on former days, and that on the whole he was better. His foot he mentioned frequently, it gave him a great deal of pain.

The rubbing in of the ℥ß of mercurial ointment had not not produced any perceptible effect upon the mouth or system.

In the evening he had just taken an opiate draught, when upon endeavouring to turn himself in bed, he was seized with a fit; he complained of his belly, but did not at the time express any sense of pain about his heart; however, he succeeded in laying himself down upon his back, and in this posture he in a few minutes silently expired without a groan, or the least appearance of a struggle.

Eleven hours after death the body was examined, while some degree of warmth was still perceptible in the viscera of the thorax.

The sternum was raised, and upon laying open the pericardium, the immediate cause of dissolution manifested itself clearly in the very remarkable condition of the heart.

The body was that of a tall man, while to the eye the heart was that of a very small person; the external form of this organ was greatly altered from its usual characters, it was in bulk so much reduced as not to occupy a fourth part of the cavity of the pericardium, in which was yet found, only the natural proportion of fluid.

The heart appeared to be very unnaturally shortened in length from the basis to the apex; the circumference of the basis was by no means diminished in the same proportion.

Upon raising up and examining the structure of the heart, its consistence and texture were found to be still more remarkably changed than its form. The resistance upon pressing the muscular ventricles, resembled that of a very firm and even horny substance, rather than of a mere fleshy cavity. The substance of the heart felt in every part unnaturally firm to a degree far beyond what can be well conceived.

The auricles as well as ventricles were greatly contracted; a section was made through the heart, by cutting across the cavities of the ventricles. The sides of the left ventricle were found in the closest contact; so much so, that the exact situation of the cavity was not immediately to be perceived.

The right ventricle, although greatly contracted, had not been able to expel the whole of its contents, about ℥ß of blood still remaining within it.

The auricle on the right side contained about ℥j. of blood, that on the left a much less proportion.

The heart was now removed from the body, and upon being being again examined a few hours afterwards, it was found to have become completely relaxed; was much larger and softer, as well as very flaccid, having entirely lost that very peculiar and remarkable state of tone which it possessed at the time of its being first inspected, when it was in the body.

The only morbid appearance found was the peculiar state of the heart above described; and thus it was on every side clear, that the muscular structure of the heart had in this case become at length subject to the same affection, which during the earlier stages of the disease was confined principally to those parts of the body subservient to voluntary motion.

[ To be continued. ]

*No. 4, Mill Street, Hanover Square.*

